# Author Correction: A cross-sectional and population-based study from primary care on post-COVID-19 conditions in non-hospitalized patients

**DOI:** 10.1038/s43856-024-00484-0

**Published:** 2024-04-01

**Authors:** Dominik J. Ose, Elena Gardner, Morgan Millar, Andrew Curtin, Jiqiang Wu, Mingyuan Zhang, Camie Schaefer, Jing Wang, Jennifer Leiser, Kirsten Stoesser, Bernadette Kiraly

**Affiliations:** 1 University of Utah Health, School of Medicine, Department of Family and Preventive Medicine, Salt Lake City, UT USA; 2 Westsächsische Hochschule - Zwickau, Faculty of Health and Healthcare Science, Zwickau, Germany; 3 University of Utah Health, School of Medicine, Department of Internal Medicine, Salt Lake City, UT USA; 4 University of Utah Health, Data Science Services, Salt Lake City, UT USA

**Keywords:** Diagnosis, Health services, Population screening, Bacterial infection, Epidemiology

Correction to: *Communications Medicine* (2024) **4**:24; 10.1038/s43856-024-00440-y, published online 21 February 2024

In this article, the affiliation details for Dominik Ose were incorrectly given as ‘2Westsächsische Hochschule—Zwickau, Department of Health and Healthcare Services, Zwickau, Germany’ but should have been ‘2Westsächsische Hochschule Zwickau, Faculty of Health and Healthcare Science, Zwickau, Germany’. The original article has been corrected.

