# Author Correction: A cross-sectional and population-based study from primary care on post-COVID-19 conditions in non-hospitalized patients

**DOI:** 10.1038/s43856-024-00661-1

**Published:** 2024-11-11

**Authors:** Dominik J. Ose, Elena Gardner, Morgan Millar, Andrew Curtin, Jiqiang Wu, Mingyuan Zhang, Camie Schaefer, Jing Wang, Jennifer Leiser, Kirsten Stoesser, Bernadette Kiraly

**Affiliations:** 1 Department of Family and Preventive Medicine, University of Utah Health, School of Medicine, Salt Lake City, UT USA; 2 Faculty of Health and Healthcare Science, Westsächsische Hochschule - Zwickau, Zwickau, Germany; 3 Department of Internal Medicine, University of Utah Health, School of Medicine, Salt Lake City, UT USA; 4 University of Utah Health, Data Science Services, Salt Lake City, UT USA

**Keywords:** Diagnosis, Health services, Population screening, Bacterial infection, Epidemiology

**Correction to:** *Communications Medicine* 10.1038/s43856-024-00440-y, published online 21 February 2024

In the originally published version of this article, the coloured squares in Figures 2 and 3 were incorrectly labelled. The orange square had a label that read ‘Patients with a positive COVID-19 test’ and it should read instead ‘Patients with a negative COVID-19 test’. The blue square had a label that read ‘Patients with a negative COVID-19 test’ and should read ‘Patients with a positive COVID-19 test’. In addition, there was an error in the caption of Figure 3, and where it read ‘Figure 3 shows the top 20 statistically significant largest differences in experienced symptoms across the severity scale (mild, moderate, and severe), in descending order by highest to lowest odds ratios’ it should read ‘Figure 3 shows the top 20 largest differences in experienced symptoms across the severity scale (mild, moderate, and severe).

