# Supplementary material for: A cross-sectional and population-based study from primary care on post-COVID-19 conditions in non-hospitalized patients
Source: Commun Med (Lond). 2024 Feb 21;4:24. doi: 10.1038/s43856-024-00440-y (PMC10881566; doi:10.1038/s43856-024-00440-y)
Supplement: Supplementary file 1 — Supplementary Information [file 43856_2024_440_MOESM1_ESM.docx]

Supplementary Figure 1: Participant Selection


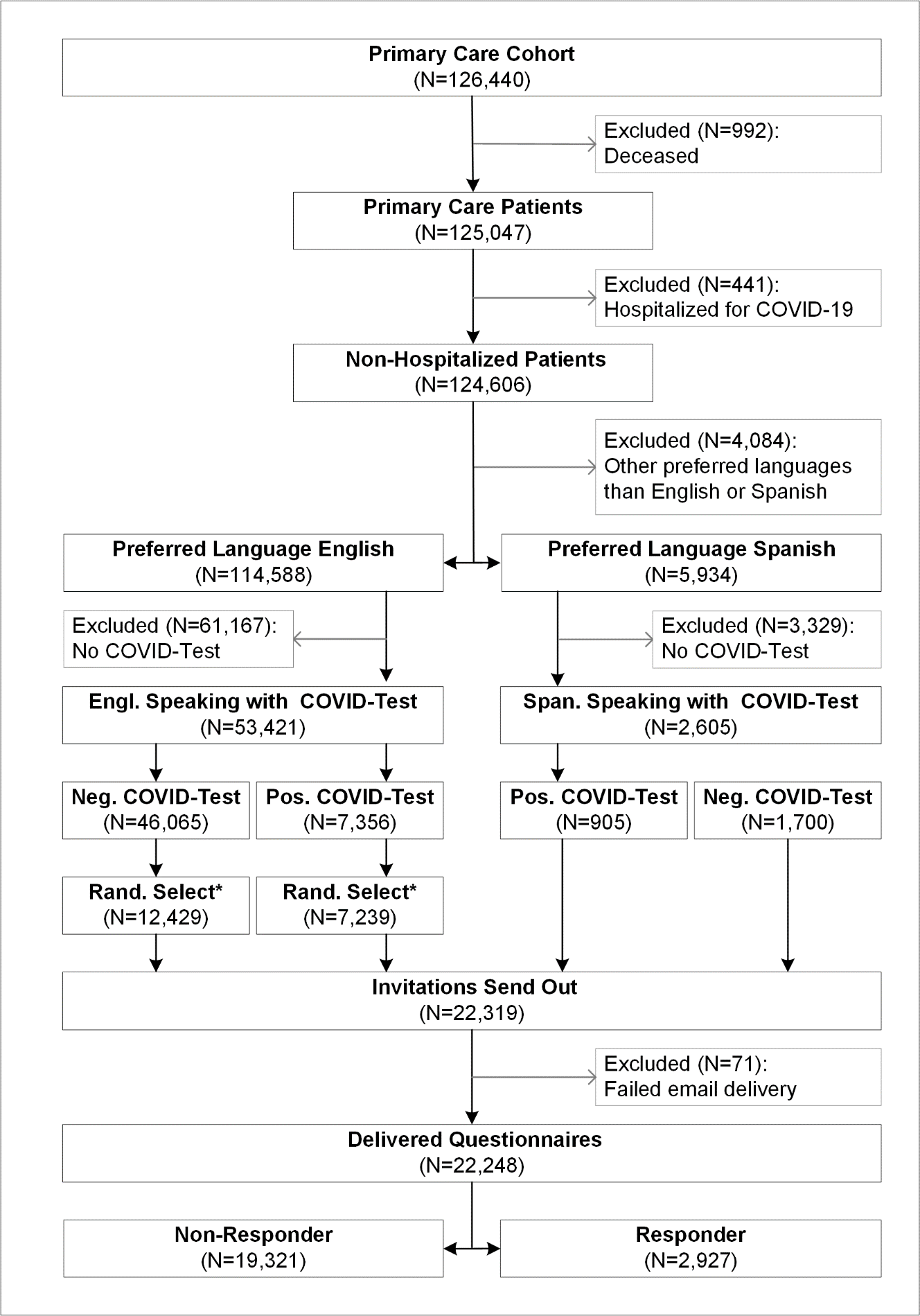


Supplementary Figure 2: Odds ratios and 95% Confidence Intervals for table 2 Frequently Reported Symptoms*****

**
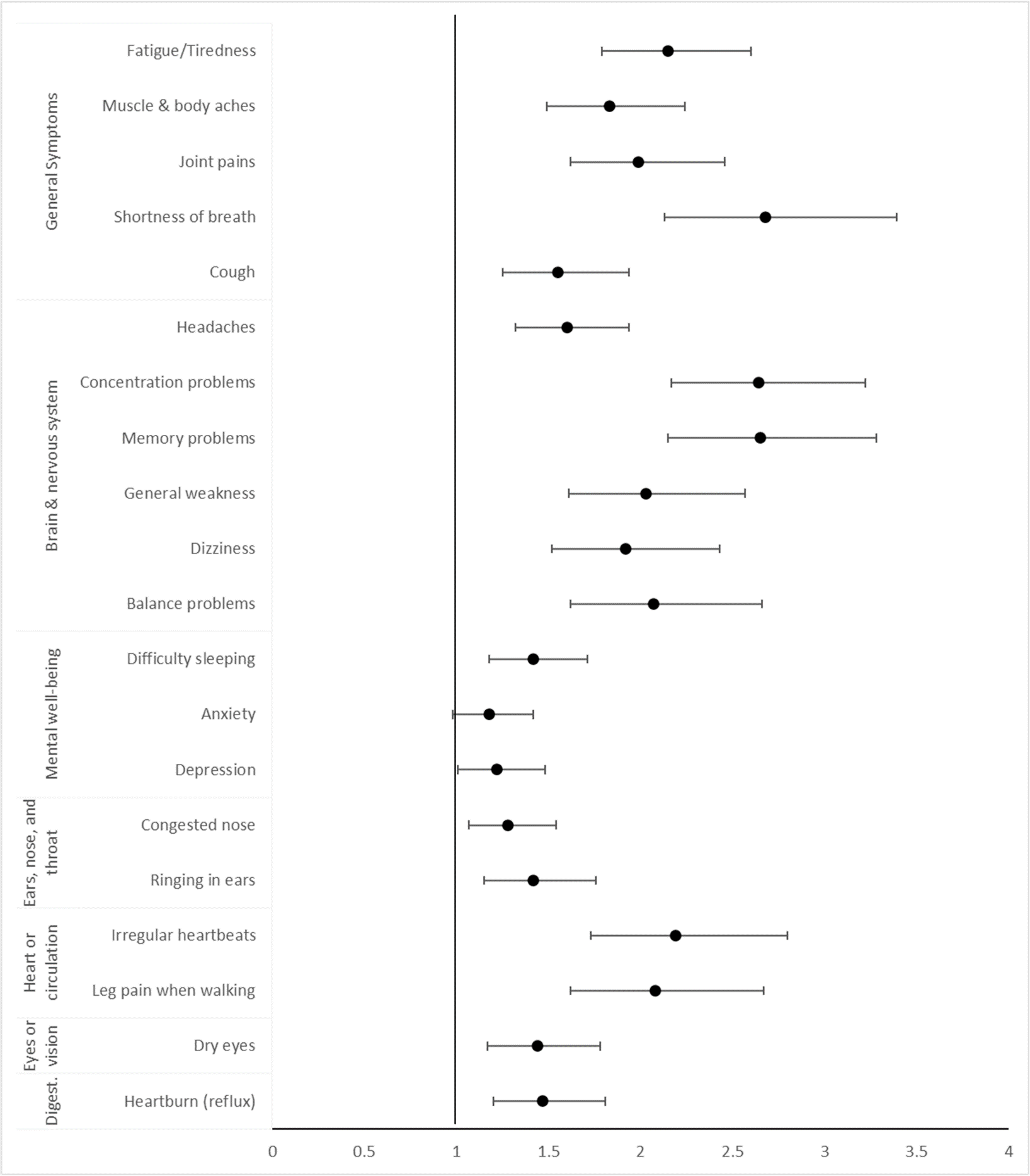
**

*Adjusted odds ratios with [95% Confidence Interval] from Table 3 shown at the end of the bars suggested relationships between COVID-19 test result and reported symptom (see Table 3 for significance p<0.05). Symptoms ordered were as listed in Table 3. Confidence intervals that did not cross the red line at 1 indicated a statistically significant association between symptom and COVID-19 test result.

Supplementary Figure 3: Frequently reported symptoms by time since COVID-19 test*

**
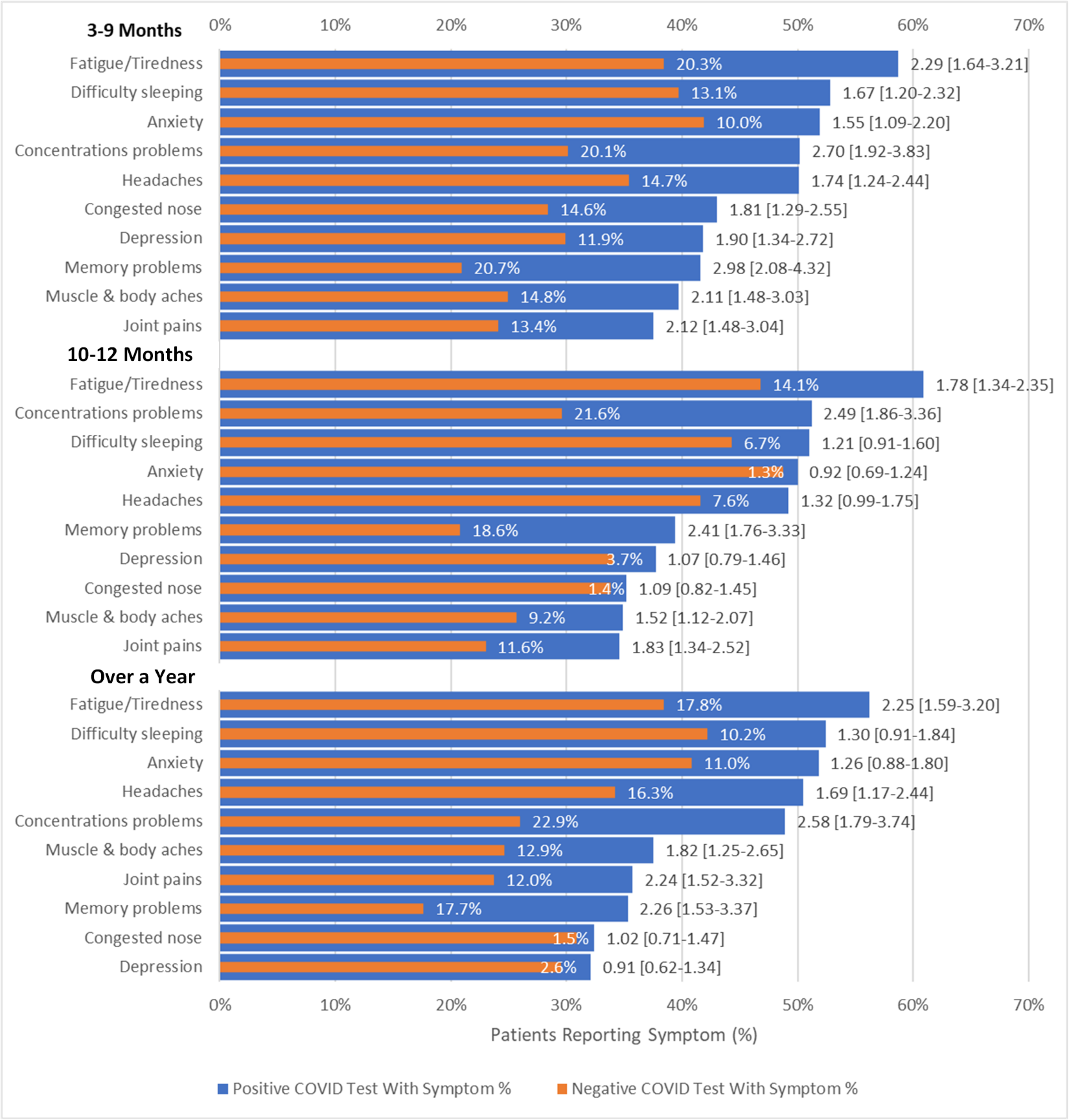
**

*Supplementary Figure 3 shows absolute differences in prevalence of top 10 most frequently experienced symptoms across the severity scale (mild, moderate, and severe) between COVID-positive and -negative patients (percentage in the white text) as well as the adjusted odds ratios with [95% Confidence Interval] comparing odds of each reported symptom between COVID-positive and -negative patients shown at the end of the bars. Absolute prevalence differences were similar for fatigue (20.3%), concentration problems (20.1%), and memory problems (20.7%), and higher than absolute prevalence differences for other symptoms. Compared to COVID-negative patients, COVID-positive patients had higher prevalence and odds of each symptom at each time point. In addition, the prevalence of each symptom (except for concentration problems) reduced over time. Depression and congested nose were not significantly associated with COVID-test results past 9 months.

| Supplementary Table 1: Symptoms, categorized by group^*^ | |
| --- | --- |
| Group name | Included symptoms |
| General symptoms | Fatigue/tiredness; chills; sweats; fever; cough; shortness of breath; wheezing; pain with urinating; muscle/body aches; joint pains; muscle twitches. |
| Ears, nose, or throat symptoms | Congested nose; runny nose; sore throat; fullness in ears; ringing in ears; hearing loss. |
| Eyes or vision symptoms | Blurry vision; double vision; dry eyes; flashes of light in vision; dark spots in vision; other vision changes; sensitivity to light. |
| Heart or circulation symptoms | Chest pain, pressure, or tightness; irregular or fast heartbeats; difficulty breathing while laying down; leg pain with walking; |
| Stomach or digestion symptoms | Diarrhea; nausea; abdominal pain; vomiting; heartburn (reflux); constipation; blood in stool. |
| Nerves and brain | Headaches; concentration problems or “brain fog”; confusion; memory problems; dizziness; balance problems; numbness or tingling; general weakness; seizures; focal weakness (limited to a specific body part); tremor (shaking in hands or body). |
| Mental well-being | Depression; sadness; anxiety; difficulty sleeping; suicidal thoughts or thoughts of self-harm; thoughts of harming others; hallucinations; nightmares; paranoia (fear that others are out to harm you). |

^*^N=54 Symptoms. Symptoms put in lay language for English and Spanish survey.

Supplementary Table 2: Non-responder analysis

|  | Population | | Responder | | | | P-Value |
| --- | --- | --- | --- | --- | --- | --- | --- |
|  |  |  | Responder^1^ | | Non-Responder | |  |
|  | N | % | N | % | N | % |  |
| Included in Analysis | 22,248 | 100 | 2,927 | 13.2 | 19,321 | 86.8 |  |
| COVID-19 Test Result |  |  |  |  |  |  | <0.001 |
| Positive | 8,757 | 39.4 | 1,667 | 57.0 | 7,090 | 36.7 |  |
| Negative | 13,491 | 60.6 | 1,260 | 43.0 | 12,231 | 63.3 |  |
| Age, mean (Standard Deviation) | 43.2 | (16.6) | 44.7 | (15.8) | 43.0 | (16.7) | <0.001 |
| Age-Categorized |  |  |  |  |  |  | <0.001 |
| 18 to 34 years | 8,190 | 36.8 | 930 | 31.8 | 7,260 | 37.6 |  |
| 35 to 49 years | 5,406 | 24.3 | 913 | 31.2 | 5,406 | 28.0 |  |
| 50 years and older | 7,739 | 34.8 | 1,084 | 37.0 | 6,655 | 34.4 |  |
| Sex |  |  |  |  |  |  | <0.001 |
| Female | 13,274 | 59.7 | 1,864 | 63.6 | 11,410 | 59.1 |  |
| Male | 8,974 | 40.3 | 1,063 | 36.3 | 7,911 | 40.9 |  |
| Ethnicity |  |  |  |  |  |  | <0.001 |
| Non-Hispanic/Latino | 13,438 | 60.4 | 2,211 | 75.5 | 11,227 | 58.1 |  |
| Hispanic/Latino | 8,286 | 37.2 | 635 | 21.7 | 7,651 | 39.6 |  |
| Unknown | 521 | 2.3 | 78 | 2.7 | 443 | 2.3 |  |
| Race |  |  |  |  |  |  | 0.001 |
| White/Caucasian | 14,373 | 64.6 | 2,333 | 79.7 | 12,040 | 62.3 |  |
| Non-White/Non-Caucasian/other | 7,281 | 32.7 | 594 | 20.3 | 7,281 | 37.7 |  |
| CCI^2^, mean (Standard Error) | 1.21 | (0.01) | 1.09 | (0.03) | 1.22 | (0.02) | <0.001 |
| Smoking Status^3^ |  |  |  |  |  |  |  |
| Never | 15,959 | 71.7 | 2,306 | 78.8 | 13,653 | 70.7 |  |
| Quit | 4,153 | 18.7 | 476 | 16.3 | 3,677 | 19.0 |  |
| Yes | 1,841 | 8.3 | 114 | 3.9 | 1,727 | 8.9 |  |
| Unknown | 295 | 1.3 | 31 | 1.1 | 264 | 1.4 |  |
| BMI, mean (Standard Error) | 30.0 | (0.06) | 29.7 | (0.16) | 30.0 | (0.06) | <0.001 |
| BMI (Kg/m^2^) |  |  |  |  |  |  | <0.001 |
| Underweight (<18.50) | 277 | 1.2 | 32 | 1.1 | 245 | 1.3 |  |
| Normal Weight (18.50-24.99) | 5,132 | 23.1 | 769 | 26.3 | 4,363 | 22.6 |  |
| Overweight (25.00-39.99) | 12,364 | 55.6 | 1,574 | 53.8 | 10,790 | 55.8 |  |
| Obese (40.00+) | 1,990 | 8.9 | 274 | 9.4 | 1,716 | 8.9 |  |
| Unknown | 2,485 | 11.2 | 278 | 9.5 | 2,207 | 11.4 |  |
| Vaccination status^4^ |  |  |  |  |  |  | <0.001 |
| None | 6,302 | 28.3 | 281 | 9.6 | 6,021 | 31.2 |  |
| Yes, Any | 15,946 | 71.7 | 2,646 | 90.4 | 13,300 | 68.8 |  |

^1^ Responder pre-exclusions. ^2^ Charlson Comorbidity Index, there were missing values of survey responses for CCI (n=4). Patient CCI Scores range 0-15. The standard error of the mean was calculated to test the mean of the sampling distribution. ^3^ Smoking status reported as “passive” treated as “yes”. ^4^ Vaccination status “yes, any” is any record or self-report of a single dose or more of any available vaccine J&J, Pfizer, Moderna, etc.

Supplementary Table 3: Largest differences between reported symptoms (n=2,539)

|  | Population | | | By COVID-19 test result | | | | | |  |  |  |  |
| --- | --- | --- | --- | --- | --- | --- | --- | --- | --- | --- | --- | --- | --- |
| Symptom |  |  |  | Positive | | | Negative | | | Difference | P-value | P-value | Odds Ratio |
|  | N^1^ | Yes^2^ | % | N^1^ | Yes^2^ | % | N^1^ | Yes^2^ | % | % | Unadjusted | Adjusted^3^ | [95% CI] |
| Concentration problems | 2,228 | 908 | 40.8 | 1,236 | 625 | 50.6 | 992 | 283 | 28.5 | 22.1 | <0.001 | <0.001 | 2.64 [2.17-3.22] |
| Memory problems | 2,226 | 677 | 30.4 | 1,233 | 486 | 39.4 | 993 | 191 | 19.2 | 20.2 | <0.001 | <0.001 | 2.65 [2.15-3.28] |
| Fatigue/Tiredness | 2,281 | 1,173 | 51.4 | 1,267 | 754 | 59.5 | 1,014 | 419 | 41.3 | 18.2 | <0.001 | <0.001 | 2.15 [1.79-2.60] |
| Confusion | 2,059 | 391 | 19.0 | 1,067 | 290 | 27.2 | 992 | 101 | 10.2 | 17.0 | <0.001 | <0.001 | 2.60 [2.01-3.40] |
| Shortness of breath | 2,272 | 527 | 23.2 | 1,261 | 384 | 30.5 | 1,011 | 143 | 14.1 | 16.4 | <0.001 | <0.001 | 2.68 [2.13-3.39] |
| Chest pain | 2,037 | 438 | 21.5 | 1,069 | 301 | 28.2 | 968 | 137 | 14.2 | 14.0 | <0.001 | <0.001 | 1.94 [1.53-2.47] |
| Blurry vision | 2,052 | 429 | 20.9 | 1,082 | 295 | 27.3 | 970 | 134 | 13.8 | 13.5 | <0.001 | <0.001 | 2.14 [1.69-2.73] |
| Muscle twitches | 2,087 | 391 | 18.7 | 1,097 | 275 | 25.1 | 990 | 116 | 11.7 | 13.4 | <0.001 | <0.001 | 2.09 [1.63-2.71] |
| Headaches | 2,215 | 977 | 44.1 | 1,224 | 610 | 49.8 | 991 | 367 | 37.0 | 12.8 | <0.001 | <0.001 | 1.60 [1.32-1.94] |
| Muscle & body aches | 2,270 | 724 | 31.9 | 1,260 | 471 | 37.4 | 1,010 | 253 | 25.0 | 12.4 | <0.001 | <0.001 | 1.83 [1.49-2.24] |
| Joint pains | 2,269 | 700 | 30.9 | 1,261 | 456 | 36.2 | 1,008 | 244 | 24.2 | 12.0 | <0.001 | <0.001 | 1.99 [1.62-2.46] |
| Irregular heartbeats | 2,242 | 447 | 19.9 | 1,243 | 311 | 25.0 | 999 | 136 | 13.6 | 11.4 | <0.001 | <0.001 | 2.19 [1.73-2.80] |
| General weakness | 2,224 | 487 | 21.9 | 1,233 | 332 | 26.9 | 991 | 155 | 15.6 | 11.3 | <0.001 | <0.001 | 2.03 [1.61-2.57] |
| Difficulty sleeping | 2,216 | 1,054 | 47.6 | 1,234 | 643 | 52.1 | 982 | 411 | 41.9 | 10.2 | <0.001 | <0.001 | 1.42 [1.18-1.71] |
| Dizziness | 2,219 | 452 | 20.4 | 1,229 | 305 | 24.8 | 990 | 147 | 14.8 | 10.0 | <0.001 | <0.001 | 1.92 [1.52-2.43] |
| Leg pain when walking | 2,239 | 421 | 18.8 | 1,241 | 287 | 23.1 | 998 | 134 | 13.4 | 9.7 | <0.001 | <0.001 | 2.08 [1.62-2.67] |
| Balance problems | 2,222 | 419 | 18.9 | 1,231 | 281 | 22.8 | 991 | 138 | 13.9 | 8.9 | <0.001 | <0.001 | 2.07 [1.62-2.66] |
| Cough | 2,263 | 525 | 23.2 | 1,253 | 330 | 26.3 | 1,010 | 195 | 19.3 | 7.0 | <0.001 | <0.001 | 1.55 [1.25-1.94] |
| Heartburn (reflux) | 2,238 | 534 | 23.9 | 1,237 | 334 | 27.0 | 1,001 | 200 | 20.0 | 7.0 | <0.001 | <0.001 | 1.47 [1.20-1.81] |
| Dry eyes | 2,238 | 585 | 26.1 | 1,242 | 349 | 28.1 | 996 | 236 | 23.7 | 4.4 | 0.019 | <0.001 | 1.44 [1.17-1.78] |

^1^ Responder (Reported yes symptom or no symptom); ^2^ N Reported patient with symptom. ^3^ P-Value calculation by Logistic Regression adjusted for age, sex, BMI, vaccine status, race/ethnicity, CCI, and time after COVID-19 test respectively.
