# Supplementary material for: A cross-sectional and population-based study from primary care on post-COVID-19 conditions in non-hospitalized patients
Source: Commun Med (Lond). 2024 Feb 21;4:24. doi: 10.1038/s43856-024-00440-y (PMC10881566; doi:10.1038/s43856-024-00440-y)
Supplement: Supplementary file 5 — Description of Additional Supplementary Data File [file 43856_2024_440_MOESM5_ESM.docx]

Description of Additional Supplementary Files

**File name:** Supplementary Data 1

**Description: – Supplementary Table 3. Supplement to Table 4: Frequently reported symptoms by age group**

**Caption: ^1^N Reported patient with symptom. ^2^Adjusted P-Value calculation by Logistic Regression adjusted for age, sex, BMI, vaccine status, race/ethnicity, CCI, and time after COVID-19 test respectively. 18-34 years as reference category. ^3^Comparison of 18-34 years to 35-49 years and 50 years and above age groups.**

**File name:** Supplementary Data 2

**Description: Supplementary Data 2 – Supplementary Table 4. Supplement to Table 8: Frequently reported symptoms by time since testing and survey receipt**

**Caption: ^1^N Reported patient with symptom. ^2^Adjusted P-Value calculation by Logistic Regression adjusted for age, sex, BMI, vaccine status, race/ethnicity, CCI, and time after COVID-19 test respectively. 3-9 months as reference category. ^3^Comparison of 3-9 months to 10-12 months and more than 12 months.**
