# Supplementary material for: A cross-sectional and population-based study from primary care on post-COVID-19 conditions in non-hospitalized patients
Source: Commun Med (Lond). 2024 Feb 21;4:24. doi: 10.1038/s43856-024-00440-y (PMC10881566; doi:10.1038/s43856-024-00440-y)
Supplement: Supplementary file 6 — Reporting Summary [file 43856_2024_440_MOESM6_ESM.pdf]

nature portfolio

Corresponding author(s): Bernadette Kiraly

Last updated by author(s): 2023/10/06

Reporting Summary

Nature Portfolio wishes to improve the reproducibility of the work that we publish. This form provides structure for consistency and transparency in reporting. For further information on Nature Portfolio policies, see our Editorial Policies and the Editorial Policy Checklist.

Statistics

For all statistical analyses, confirm that the following items are present in the figure legend, table legend, main text, or Methods section.

n/a	Confirmed	
☐	☒	The exact sample size (n) for each experimental group/condition, given as a discrete number and unit of measurement
☐	☒	A statement on whether measurements were taken from distinct samples or whether the same sample was measured repeatedly
☐	☒	The statistical test(s) used AND whether they are one- or two-sided<br>*Only common tests should be described solely by name; describe more complex techniques in the Methods section.*
☐	☒	A description of all covariates tested
☐	☒	A description of any assumptions or corrections, such as tests of normality and adjustment for multiple comparisons
☐	☒	A full description of the statistical parameters including central tendency (e.g. means) or other basic estimates (e.g. regression coefficient) AND variation (e.g. standard deviation) or associated estimates of uncertainty (e.g. confidence intervals)
☐	☒	For null hypothesis testing, the test statistic (e.g. F, t, r) with confidence intervals, effect sizes, degrees of freedom and P value noted<br>*Give P values as exact values whenever suitable.*
☒	☐	For Bayesian analysis, information on the choice of priors and Markov chain Monte Carlo settings
☒	☐	For hierarchical and complex designs, identification of the appropriate level for tests and full reporting of outcomes
☒	☐	Estimates of effect sizes (e.g. Cohen's d, Pearson's r), indicating how they were calculated

Our web collection on statistics for biologists contains articles on many of the points above.

Software and code

Policy information about availability of computer code

Data collection	Secondary data on clinical characteristics of participants (primary care patients) were obtained from the University's Enterprise Data Warehouse (EDW). Post-COVID-19 symptoms were self-reported by participants through questionnaires in REDCap with their consent.
Data analysis	Descriptive statistical analysis included the Chi-Square test and two-sample t test. Inferential analysis included logistic regression with the Tukey's multiple comparison test. All analyses were conducted in R Studio.

For manuscripts utilizing custom algorithms or software that are central to the research but not yet described in published literature, software must be made available to editors and reviewers. We strongly encourage code deposition in a community repository (e.g. GitHub). See the Nature Portfolio guidelines for submitting code & software for further information.

Data

Policy information about availability of data

All manuscripts must include a data availability statement. This statement should provide the following information, where applicable:

- Accession codes, unique identifiers, or web links for publicly available datasets
- A description of any restrictions on data availability
- For clinical datasets or third party data, please ensure that the statement adheres to our policy

Data are available upon request to the scientific community upon submission of a data request application via the corresponding author.

Human research participants

Policy information about studies involving human research participants and Sex and Gender in Research.

Reporting on sex and gender	Sex was defined as a biological variable to describe biological differences and influences when comparing males and females. Thus, sex was coded binary as male or female as opposed to gender, which could exist on a spectrum and is more often used when describing social or psychological differences between men, women, and other genders. Sex was defined in the main text on page 7 and reported in Tables 2 & 5 and Supplementary Table 1.
Population characteristics	Outcomes: Common post-COVID-19 symptoms were self-reported by both COVID-19 positive and negative patients in their questionnaires, classified into 7 categories, including 1) general symptoms (fatigue/tiredness, muscle & body aches, joint pains, shortness of breath, cough); 2) brain & nervous system headaches (concentration problems, memory problems, general weakness, dizziness, balance problems); 3) mental well-being (difficulty sleeping, anxiety, depression); 4) ears, nose, and throat (congested nose, ringing in ears); 5) heart or circulation (irregular heartbeats, leg pain when walking); 6) eyes or vision (dry eyes); 7) stomach or digestion (heartburn).<br><br>Exposure: The secondary COVID-19 test result (positive, negative) for each participant was documented in the EDW. In addition, participants could self-report a positive COVID-19 test result outside the University of Utah health system. If they did, the answers were included when defining COVID-19 test result.<br><br>Covariates: Information on the following secondary demographic & clinical characteristics was obtained from the EDW. Sex was defined as a binary biological variable to describe biological differences and influences when comparing males and females. Other covariates included age (18-34 years, 35-49 years, 50 years and above), race (American Indian/Alaskan Native, Asian, Black/African American, Native Hawaiian/Other Pacific Islander, White, Other, Unknown), ethnicity (Hispanic/Latino or non-Hispanic/Latino), BMI (<18.50: underweight, 18.50-24.99: normal weight, 25.00-39.99: overweight, 40.00+: obese), smoking status (never smoked, quit smoking, currently smoking), COVID-19 vaccine status (none, any dose), time between testing and questionnaire receipt (3-9 months, 10-12 months, more than 12 months), and Charlson Comorbidity Index (Scored 0-15).
Recruitment	We used the university's EHR database to identify 126,440 primary care patients. Of those primary care patients, 124,606 were not hospitalized for COVID-19. After using the inclusion/exclusion criteria, we included 22,319 non-hospitalized patients who spoke English or Spanish with COVID-19 test results. Invitations were successfully sent to 22,248 patients for completing the questionnaire on post-COVID-19 symptoms. Finally, 2,539 patients completed and submitted the questionnaire with self-reported common post-COVID-19 symptoms. Detailed information about the recruitment was provided in the flow diagram in Supplementary Figure 1 in Supplementary Information.
Ethics oversight	The University of Utah Institutional Review Board (IRB #139714) exempted the study because there was no more risk than expected posed on participants for completing the questionnaire (e.g., sensitive questions related to mental health) in this observational study.

Note that full information on the approval of the study protocol must also be provided in the manuscript.

Field-specific reporting

Please select the one below that is the best fit for your research. If you are not sure, read the appropriate sections before making your selection.

☒ Life sciences ☐ Behavioural & social sciences ☐ Ecological, evolutionary & environmental sciences

For a reference copy of the document with all sections, see nature.com/documents/nr-reporting-summary-flat.pdf

Life sciences study design

All studies must disclose on these points even when the disclosure is negative.

Sample size	Given the sample of 2,539 participants (1,410 COVID-19 positive, 1,129 COVID-19 negative) who reported common post-COVID-19 symptoms, power analysis was conducted to calculate power for the hypothesis test for a significant difference in prevalence of post-COVID-19 symptoms between COVID-19 positive and negative patients. Using a two-sided Chi-square test at a significance level of .05, we would have 93%-99% power to detect a small effect size of 0.15-0.2.
Data exclusions	We used the following participant inclusion criteria: Age 18+, at least one prior visit (in-person or virtual) with a U of U Health primary care center between January 2020 and March 2021, email address on file, preferred language English or Spanish, and a positive or negative COVID-19 test result (PCR) between March 1st, 2020, and August 31st, 2021, documented in their electronic health records (EHR). Patients were excluded if they had a COVID-19 test before March 1st, 2020, or if they were hospitalized or sought emergency department care related to COVID-19.
Replication	NA because the study is observational.
Randomization	NA because the study is observational.

Blinding	NA due to the use of a cross-sectional design.

Reporting for specific materials, systems and methods

We require information from authors about some types of materials, experimental systems and methods used in many studies. Here, indicate whether each material, system or method listed is relevant to your study. If you are not sure if a list item applies to your research, read the appropriate section before selecting a response.

Materials & experimental systems

n/a	Involved in the study	
☒	☐	Antibodies
☒	☐	Eukaryotic cell lines
☒	☐	Palaeontology and archaeology
☒	☐	Animals and other organisms
☒	☐	Clinical data
☒	☐	Dual use research of concern

Methods

n/a	Involved in the study	
☒	☐	ChIP-seq
☒	☐	Flow cytometry
☒	☐	MRI-based neuroimaging
